# Reviews and Book Notices

**Published:** 1879-09

**Authors:** 


### Reviews and Book Notices.

ARTICLE XVII.—THE REPORT OF THE DEPARTMENT OF HEALTH OF THE CITY OF CHICAGO, FOR THE YEAR 1878.

The recent appearance of this annual document suggests a number of interesting reflections. A considerable portion of the report, aside from the purely statistical matter, is occupied by an account of the investigation of trichina in pork, conducted by Mr. H. F. Atwood, vice-president of the State Microscopical Society, and Dr. W. T. Belfield, Demonstrator of Physiology in Rush Medical College. Out of one hundred hogs examined by these gentlemen, eight per cent. were infested by the parasite. They, however, were able by their experiments to confirm the opinion of previous investigators, that a temperature less than that of boiling water is sufficient to destroy the trichina, so that cooked pork need not be feared. Dr. Belfield went still farther. He swallowed a piece of fresh meat in which the microscope exhibited twelve living worms, but he has never experienced the slightest disagreeable result. It is thus rendered highly probable that a considerable dose of the parasite is needed to produce sensible toxic effects.

The subject of regulating offensive trades forms a very interesting section of the report. The health commissoner has favored us with a full account of the manner in which the "143,227,070 pounds of blood, bones, intestines, scraps, etc., were furnished by our own slaughtering houses, to be cooked and dried for market during the year past." The careless manner in which this was done has been the cause of the fearfully offensive odors which for years have disturbed the comfort of our citizens. The commissioner gives a very graphic account of the difficulties which were encountered in the effort to abate this nuisance. The final triumph of the officials who were charged with this matter, shows what can be accomplished when energetic legal measures are based upon sound moral principles. No man has a moral right to deliberately create and maintain a nuisance in the face of his fellow men. Planting itself upon this high and unassailable position, the Supreme court of the State amply vindicated the rights of the people, and permitted no petty quibbles to hinder the operation of the measures of self defense for which we are indebted to the ex- city attorney, Hon. Richard H. Tuthill. It is, however, to be regretted that from this gallant conflict the officials did not emerge with hands thoroughly clean. The evil which they attacked was a nuisance of the most offensive character, but there is not the slightest shadow of evidence that it was dangerous to the health of the city. In their zeal against the enemy, the officials did no little harm to the community by painting the foe in colors darker than could be justified by the facts. Such stenches are very disagreeable, but it has never been shown that they produce or even aggravate diseases. To say nothing of considerations drawn from chemistry and physiology, the mortality reports of the city are a sufficient proof of this proposition. With an annually increasing production of stenches there has been a continually decreasing rate of mortality. We have no doubt that many delicate persons have experienced an increase of suffering in consequence of reading and hearing so much about the dangerous character of the slaughter-house gases. For this the slaughter-houses are not so much to blame as the ignorance or the zeal which propagated such errors.

The brief remainder of the report is occupied with a variety of subjects, first of which may be noticed the returns of births and deaths, from which it appears that the number of deaths during the year 1878 was 7,422. "The total death rate to population was for the year 16.50 per 1,000;" 11,152 births were registered during the year. The commissioner rightly believes that this figure still falls "short of the actual number of children born in our city." He also volunteers the statement that "the legal value of such registration is of vast importance, and the humblest born child has rights in this matter of which our profession is made the guardian." Now, if it is true that the value of such registration is of vast importance, and if the humblest-born child has rights in this matter, there is no one to whom the care of such valuable rights more properly belongs than to the legal and natural guardians of the child, its parents. If the law-makers hold the opinion that the protection of this right should be taken from those who are most interested in its security, and should be assigned to strangers who have not the slightest interest in the matter, it is worth while at least to discuss the wisdom of trying to secure the desired result by police regulations which can be evaded without difficulty, and which, moreover, are in direct antagonism with that article of the State constitution which declares (Art. II, Sec. 13) that "private property shall not be taken or damaged for public use without just compensation." We are very well aware that the world is full of people who believe in making doctors work for nothing; but certainly no reputable physician, even though he be the tenant of an official position, should ever countenance such injustice. Every law, moreover, which, like this, diminishes the responsibility of parents by transferring a portion of their duty to the shoulders of strangers who are reluctant to assume such care, is a positive injury to the entire community. Good laws seek to foster the sense of individual responsibility where it belongs. Bad laws diminish that sense, by transferring to the government, or to certain classes, those duties which pertain to every citizen in his private capacity. In pleasing contrast to the sullen brutality with which our new State law demands uncompensated pecuniary outlay and service on the part of physicians, is the recent action of the United States government in connection with the approaching national census. A polite note from the superintendent of the census invites, but does not command, a return of deaths from ever physician. The necessary forms are furnished, post paid each way. If any additional expense should be incurred, it will be promptly refunded by the government. Such a request cannot fail to meet a cordial response, even from men who would go to prison rather than yield to a forcible invasion of their constitutional rights.

A brief paragraph is all that is devoted to scarlet fever. The report states that "placing of warning cards still receives the almost unanimous approval of the profession in the city"—an assertion sufficiently robust to rejoice the most ardent admirer of the late Baron Munchausen. The fact is, that the profession has learned to "get around" the law so comfortably, that its present administration is no longer the nuisance which it once was. But, even if the profession were unanimous in their approval of warning cards, that would not make it right for the government to intrude them into the private abode of private citizens. The warning card, as a notice of sickness, is in itself harmless enough, but as the symbol of an officious intrusion by the government, it is thoroughly outrageous. The officials may issue advice, and may properly recommend the use of cards, if they must do something; but when they go beyond this limit, they become impertinent, offensive, and injurious to the cause of sanitary improvement, as the frequence with which scarlet fever is successfully concealed abundantly proves. In dealing with this subject, the officials need not wait for orders from Boston, but they should learn wisdom from the course of the intelligent physician, who, when people refuse to be drenched with Glauber's salts, asks himself whether a bottle of citrate of magnesia or a sugar-coated pill will not do the work as well. Doctors who insist upon forcing nauseous doses into their patients need not be surprised if common folks keep away, and do not let them know when they are sick—especially after they have learned that the filthy drought does no good. Health officers may very properly *advise* such measures, if they consider them useful; but by legislative enactment to *compel* the posting of warning cards and the private performance of other sanitary operations which may chance to be fashionable, is a great mistake. Sanitary science, so-called, is in a very crude condition, and much that is now thought essential—remedies which enthusiasts are anxious to ram down the throats of everybody—will soon have to be abandoned, because in direct opposition to those higher truths of science which are at present known to comparatively few, but which will become the common property of a subsequent generation. It is a very difficult thing to repeal even a bad law; consequently it is unwise to give the authority of law to the shifting opinions of a half formed science. Already one consequence of erroneous teaching and unscientific law-making is apparent in the growth, inside as well as outside of the profession, of a morbid public sentiment regarding the nature and functions of disease, which will greatly retard the recognition of true science. Until the fundamental principles of science, and the highest results of experiment and observation become more generally known to sanitarians, and to the medical profession, health officers should be very cautious about threatening people with the terrors of the law. It is too much like trying to create an established church by the aid of dungeon and faggot. For a long time yet, the principal occupation of our officials should be the search for truth and the diffusion of genuine knowledge, rather than the enforcement by law of questionable dogmas based on doubtful hypotheses of the most transitional character.

The proceedings of the Health Office relative to small-pox and yellow fever scarcely call for comment. We may, during the prevalence of the imported epidemics — cholera, small-pox, yellow fever, typhus fever and the plague — cheerfully applaud the very methods which are utterly wrong in the conduct of the government towards those ordinary endemic diseases for which the ordinary resources of the community have always sufficed. As in time of war we praise the general who rides rough-shod over ranks of living men, with no thought of anything but victory ; so in times of imported epidemic disease, the officials may exercise a license which no common endemic can ever excuse. The great fault of our officials consists in the fact that they have not yet learned in times of peace to refrain from martial practices. A conspicuous example of this common defect is found in the following declaration of the report (p. 9) : "To reform tenement houses, suppress epidemics of preventible diseases, reconstruct, clean and disinfect vaults, regulate offensive trades, inspect the food supplies, and destroy or consign to the rendering tanks everything unwholesome, to elevate the standard of municipal cleanliness in streets and alleys and yards — all this is the legitimate work of the Health Department, and concerns every citizen." It would be difficult to find a better illustration of that confusion of ideas which is so common among inexperienced sanitarians who have not yet mastered the alphabet of sociology and of political science. Because it may be proper for public officials to keep the streets and alleys clean, it does not follow that it will be wise for them to "reform tenement houses, and reconstruct vaults." Because it may be expedient that they should maintain quarantine against foreign plagues, it does not follow that they can wisely or successfully attempt to "stamp out" endemic diseases which are most efficiently treated by private enterprise. The utterance of the Commissioner amounts to nothing more nor less than the adoption of the entire Communist programme, which throws upon the State the whole responsibility for the care and maintenance of every citizen. It, moreover, attempts to justify the importation of rude, despotic methods into the quiet repose of civil life. This reckless firing of revolvers and slashing with sabers among inoffensive citizens — this cheerful willingness to blow up houses, with people in them, when there is neither war nor conflagration to justify such violence — this stentorian roaring for a fire-engine to put out a bedroom candle — is what fills thoughtful men with disgust at many of the antics of our sanitary officials. Nor can the results of their efforts be urged in excuse for such unnecessary intrusion. A candid review of the effort to regulate tenement houses in London, or the social evil in St. Louis, is sufficient to show the utter failure of such official intermeddling. To get at the facts in such matters, it is necessary, however, to seek information from other sources than the proverbially one-sided and self-laudatory official documents. Poverty constitutes no valid reason for interference with the domestic affairs of people. The instincts of even the poorest classes prompt them, like other people, to live in houses as convenient and as healthful as they can afford. Compulsory sanitation only creates additional expense, which drives the poor into still poorer quarters, so that they cannot be helped in that way. The only method that has thus far proved successful in every sense of the word, is the method of private benevolence in the way of disseminating advice and the means of moral improvement among the lower classes. No amount of legislation will ever reach the difficulty ; but every increase of knowledge, and every advance in moral evolution, will proportionately relieve these difficulties, without the aid of civil law.

It is frequently stated that the poor should be protected by the government against the causes of disease which are said to infest the habitation of the lower classes. It is true that certain diseases are more often encountered among the poor than among the rich, but the reverse of the proposition is also true. Every class of people has to contend with its own peculiar difficulties; and if it is right for the government to assume the special protection of any one class, there is no reason why it should not equally assume the protection of all. But the intelligent and energetic members of society prefer to provide for themselves in all the ordinary exigencies of life. It is the lazy people and their sentimental friends who are always calling for government aid. If now you undertake to protect this fraction of the community, you have to protect it against the consequences of idleness, luxury, intemperance and vice — thus interfering with the operation of the wholesome monitory laws of nature; and you do it at the expense of the meritorious classes of society. Having thus accustomed such worthless people to rely upon the government for protection against small-pox, and scarlet fever, and syphilis, and diphtheria, and sewer-gas, and scabies, it will not need the passage of many generations before they will demand protection by the government against the cold and hunger and nakedness for which they should themselves make provision. They will even demand to be washed at the public expense; and they will have no trouble in finding some demagogue ready to tax me heavily, that he may enjoy the advantage of running a free bath-house for the comfort of ragamuffins and black-legs whose only merit consists in their ability to handle a ballot on election days. It is even proposed to dignify such robbery by the name of *solicitude for the public health!*

This is the way in which Communists are produced; and a government which adopts the protective policy may thank itself alone for the growth of Communism and Socialism among its citizens.

The only wise course for society to adopt in relation to the evils which weigh upon different classes of people, is to undertake their relief by private benevolence. An organized distribution of private charity, combined with a similar diffusion of scientific information and moral education, among the ignorant classes who form the extremes of society, would accomplish far better results than can ever be effected by the largest army of politico-sanitary officials. A comparison of the work done by a philanthropic organization, like the Chicago Relief and Aid Society, with the mysterious performances of the County Agent, will sufficiently illustrate this point.

The only remaining portion of the report which demands notice is the section which treats specifically of tenement-houses. These are arbitrarily defined as dwellings in which more than three families keep house independently. The Commissioner finds this an almost unlimited "field of sanitary work." Finding no legal authority "to order the vacation of uninhabitable buildings, and to refuse to let them be again occupied until placed in proper sanitary condition," he has availed himself of the provisions of the following legislative enactment of March 9th, 1867: "In case the sanitary condition of the city should be of such a character as to warrant it, it shall be the duty of the Commissioner of Health to take such measures, and to do and order, and cause to be done, such acts for the preservation of the public health (though not herein or elsewhere or otherwise authorized) as he may in good faith declare the public safety and health to demand."

Taking this legislative utterance as his authority, he has undertaken to interfere, at his own discretion, with the natural laws of supply and demand which operate in tenement houses as well as everywhere else. This arbitrary assumption of extraordinary powers, for the purpose of dealing with the ordinary conditions of the daily existence of society, has been formally condemned by the highest courts of appeal. In New York the Board of Health sought to justify their analogous action in the matter of night scavenging by an exactly similar appeal to authority which was by law designed to be invoked only during extraordinary emergencies. The case was fully discussed in the courts, and the arbitrary action of the Board of Health was formally condemned by the Court of Appeals (Gregory vs. New York, 40 N. Y. 273). A dim consciousness of error seems to have invaded the mind of our Commissioner, for he goes on to say: "It is true that this act was drawn to meet such emergencies as epidemics may present, but it is also true that these domiciles, without the energetic and vigilant attention of sanitary authority, become nurseries of every form of contagious disease, and of perpetual epidemics, and thus always stand as a great menace to the public safety and health" — all of which, by the way, is a gross exaggeration, which merits the sharpest rebuke.

Here, then, we have a public official openly confessing the commission of one of the crying sins of the age. Having no legal sanction for certain action, he nevertheless goes right on to act in accordance with his own arbitrary notions, and seeks to justify this course of behavior by an appeal to a law which he himself admits was enacted to cover a state of affairs which has at present no existence. That is the Turkish way of carrying on a government, but it is not a civilized mode of proceeding. This readiness to act without authority of law — this fondness for autocratic and mediæval methods of administration — constitutes one of the standing grounds of complaint against our modern sanitary officials. They delight in vague and general legislation which defines little, and leaves almost everything to the discretion of an officer who can throw dust in the eyes of the public by loose talk concerning the exigencies of the public health, whenever he is taken to task for unnecessary acts of despotism. Now such a state of things is all wrong. The conditions of public health are as simple and as capable of clear definition as the conditions of commerce or of agriculture. There is no more need of giving arbitrary powers to a Health Commissioner than there is of giving to a Custom-house officer the privilege of collecting such duties as he may see fit to impose. There is great need of improvement of our sanitary laws in this particular. They have evidently been drawn up with very little knowledge of the proper functions of government, and with a very imperfect comprehension of what should be done, or left undone, by the health officials. Consequently, while very minute in their specification of certain minor duties — such as registration of births and deaths — they are virtually silent about all the higher and more important duties of officers who are charged with the public health. Nothing could be more gratifying to the ordinary politician; nothing more disgusting to the scientist and the philosopher. What we need, in the first place, is a clear understanding of the sphere and legitimate functions of government in general. Then we need a complete definition of the limits which divide the public and common interests of men from their private interests and relations. Finally, we need a thorough recognition of what constitute the ordinary conditions and necessities of a community, and of what are the extraordinary and, so to speak, accidental exigencies of such a society. These elements of the problem are not difficult to define. But when we attempt to adapt legislation to these conditions, it becomes painfully evident that very, very few of our legislators are yet possessed of the knowledge and wisdom and moral sensibility necessary to reach a correct result. Take, for example, such a little trifle as the question whether the highest good of the community will be secured by placing warning cards upon infected houses, as was anciently done in epidemics of the plague, and as we now sometimes do in scarlet fever. The ordinary sanitarian, who thinks only of the avoidance of a *contagium*, jumps at once very naturally to the conclusion that a warning card and a stringent quarantine will accomplish the best possible result. But when we examine the subject calmly, in accordance with the clearest light that radiates from the triune source of science, philosophy and moral law, we find that the first thought of the ordinary sanitarian was erroneous, and that the measures founded upon his hasty conclusions not only defeat themselves, but also work positive injury to the human race. This, no doubt, seems like a very startling assertion; and, at the present stage of the evolution of the average of mankind, it will not command anything beyond a very limited acceptation. In fact, we should need the space of an essay for the adequate presentation of the highest scientific thought regarding these subjects. If, however, any person of ordinary cerebral capacity desires to reach a definite and final conclusion regarding these matters, he can do so, but in only one way. He must first thoroughly master the principles of physics, of chemistry, and of physiology. (We may remark, as we go, that this will require something more than a mere recollection of one's slumbers upon the benches of a medical college). He should then carefully read the works of the masters in biology. A complete study and practical knowledge of medical science should then follow. After this the advancing student should endeavor to compass the field of mental and moral philosophy. Then should come an investigation of the principles of law and of government; and lastly, we may conclude with a wide range of historical reading. Thus equipped, it is not difficult to judge of the right or the wrong of any question which may present itself to the sanitary legislator. Such a course of training is by no means beyond the reach of ordinary mortals — it is indeed the possession of a great many comparatively unknown individuals. But the attributes which carry one through such a course of training are not often united with the qualifications which win success in the political arena; consequently, the ordinary sanitarian, having become a sanitarian through the force of political combinations, finds himself incapable of accurate independent determination, based upon knowledge of scientific facts. Like all persons similarly situated, he either abandons himself to the blind guidance of an arbitrary will, or else he stands feebly agape, waiting for the utterances of some little sanitary pope in some distant quarter of the earth. The greater the distance, the greater the authority which, for him, invests such deliverances; hence one of the causes which give such currency to the Asiatic method now so fashionable.

We have dwelt thus at length upon this report, because it embodies nearly all of the fundamental errors which are now current in official circles. These errors are based upon doctrines of which the inaccuracy is well known to the higher circle of scientific men. But knowledge trickles very slowly through the masses of mankind; consequently, we must wait with patience until our sovereigns can learn. Fortunately, however, the general drift is in the direction of progress, and the time will surely come when this little flurry of awakening ignorance will have ceased, and our children will study with amusement the history of the manner in which we had to deal with this attempted revival of exploded ideas from the primitive ages of a barbarous world. The crudity and immaturity of thought which characterizes the half-educated sentimentalists who are running to and fro, disturbing everybody with their old-fashioned whims, is well illustrated by their favorite maxim, "Health is more important than civil liberty." That is, they believe in treating the human race, not like free and intelligent moral beings, but like so many brute cattle, who are to be fed and watered and salted and branded and castrated according to such notions as may be fashionable among the tiny despots who are from time to time endowed with a little brief authority over the uncomplaining herd. That was the universal theory of government during the dark ages; but the more enlightened portions of the world are gradually outgrowing such ignoble ideas

And now, in conclusion, let us not be misunderstood. In all this criticism we have no personal feelings to indulge. We speak only in behalf of true science and good government. While we must denounce error and cant and folly wherever they exist, we should thoroughly believe in sanitary laws, and in a stringent sanitary administration. But the laws must be wise, and their administration must be just. Our officers deserve great credit for much that they have done. For the conclusive manner in which they have demonstrated the impossibility of stamping out scarlet fever; for the splendid energy with which they have desolated Bridgeport; for the scientific way in which they have ventilated the sewers; for the general cleansing of streets and alleys; and for all similar beneficent actions, they deserve the highest praise. It is only when they have allowed themselves to be led astray by fallacies which are fashionable from Berlin to San Francisco, that they have exposed themselves to the shafts of an impartial and scientific criticism. H. M. L.

ARTICLE XVIII.—HABITUAL DRUNKENNESS AND INSANE DRUNKARDS. By Dr. Bucknill, F.R.S., Fellow of the Royal College of Physicians—Late Lord Chancellor's Visitor of Lunatics.

In 1875, Dr. Bucknill visited the United States, and, being interested as a mental physician in drunkenness as one of the great factors of insanity, made numerous inquiries in regard to the results attained in inebriate asylums and homes of this country.

The doctor associated with and interviewed many of our oldest superintendents of institutions for the insane, and visited all the inebriate asylums and homes he could hear of in the Eastern States, except "one small private institution." There is no evidence whatever that has come to the reviewer's notice which shows that Dr. Bucknill was not perfectly honest in all his inquiries, or that they were made with any other purpose than that of eliciting truth. He does not believe in the pernicious doctrine that habitual drunkenness is a disease; therefore he was very severely criticised by those interested in the establishment of that dogma.

Sometime after his return to England — indeed, the following year — in a speech before the Rugby Temperance Association, he took occasion to state his views in regard to intemperance, and expressed his opinions candidly as to the danger of calling habitual drunkenness a disease. He also spoke in unmistakable terms of the direful effects of treating men for a disease which did not exist, by remedies which were not applied.

He says at p. 3, Art. III. that "he believed drunkenness to be a fruitful cause of disease, but not in itself a disease; and he looked upon inebriate asylums as an unfortunate attempt to coddle drunkenness and patch up a wide and fruitful social mischief."

In all his remarks, however, Dr. Bucknill excepts the Franklin Reformatory, of Philadelphia — of which Dr. R. P. Harris is physician — and it should be remembered that he did not visit the Washingtonian Home of Chicago.

Both these institutions have been ignored by the American Association for the Cure of Inebriety, because they refuse to inscribe on their banner, "Intemperance is a Disease."

Concerning the Franklin Home, he says: "This was the only place I saw in America where honest, earnest work was being done, not for the CURE but for the REFORM of inebriates." The doctor was very greatly pleased with the spirit manifested by the inmates of this Home, and expressed the desire to see institutions of this order established in Glasgow and Liverpool.

In regard to the heredity of intemperance, our author speaks with care, and with the evident desire of giving it all the prominence which the facts will justify. I am hopeful that his words will influence some of those who so hastily jump at the conclusion that the appetite for alcohol, in any considerable number of cases, is handed down from a former generation, thus doing away with all personal responsibility in the matter. As a matter of fact, many of those whose reformation is the most easily accomplished, and the most perfect as regards time, are those whose ancestors, for two generations back, have been addicted to the habitual use of alcoholics; while on the other hand some of the most incorrigible — those who, after repeated admissions, and upon whom months of personal labor have been bestowed to insure a reformation, relapse immediately into their former habits — come from a stock perfectly free from the use of stimulants.

At p. 34 is found the following: "I am inclined to think that heredity from intemperance is a less important factor of insane drunkenness than it is generally supposed to be;" and still further he says: "However influential in the conduct of life a truth may be, however wholesome its full force, it is morally wrong and practically mischievous for it to be overstated, which I fear has been done with regard to the heredity of drunkenness. Moreover, if it be admitted that the tendency to drink is transmitted from one generation to another, and that the children's teeth are set on edge because the parents have eaten sour grapes, it does not prove that such an inherited tendency is morbid, for vice also is heritable."

We have here a book which every man and woman interested in the great work of reforming inebriates, should read. Doctors, lawyers and clergymen should read it. Above all, editors, philanthropists and temperance lecturers should buy a copy.

The work contains 100 pages, and is the best argument yet published against that pernicious dogma, that "drunkenness is a disease."

It shows with clearness and conclusion that reformation is something more than "leading for a brief time a life of indolent luxury, under a cloud of constant tobacco-smoke, with cards and billiards," and being impressed at the same time that they (men addicted to habitual drunkenness) are "interesting but helpless objects of social and psychological science."

Since 1873 (*Medical Examiner*, Chicago), the writer of this review has advocated the theories now so ably given to the public by Dr. Bucknill, and it is to be earnestly hoped that the time is not distant when every right-thinking and right-feeling individual will use his influence against the mischievous doctrines promulgated by many so-called reformers, but which are so fully exploded in the pages reviewed. C. W. E.

Article XIX. — Demonstrations of Anatomy; being a Guide to the Knowledge of the Human Body by Dissection. By George Viner Ellis. From the eighth English edition; pp. 716. Philadelphia: H. C. Lea.

This is the latest edition of a work well known in this country as well as in England. It is exactly what it purports to be — not a comprehensive manual of anatomy, but a practical guide for the worker, containing full and explicit directions, equally valuable to the novice or the advanced student. Inasmuch as it covers the same ground as Holden's somewhat less pretentious work, it is natural to compare the two. While hardly more practical, it is more full, containing probably the equivalent of two hundred pages of matter more than the latter.

If we complain at all, it must be of the illustrations in which reference is made to different parts by letters or figures, necessitating more time and possible confusion in identification, and being rather wearisome to the eyes. While some of the woodcuts are rather crude, a good feature of the work is the repetition of the same cut to avoid turning back a number of pages.

On the whole, we commend the work as probably the most complete of its kind in our language, adapted to the needs of all, but containing for the average student, perhaps, an embarrassment of riches. R. P.

Article XX. — A Practical Treatise on Surgical Diagnosis. By Ambrose L. Ranney, A.M., M.D.; pp. 386. New York: Wm. Wood & Co.

The general impression left after examining this work is favorable. We hold that the general form in which the salient points of different affections can be best comprehended and contrasted, is the one which the author has adopted. There is nothing strikingly original about the work, nor can it supply the place of larger treatises on surgery, but it certainly sets forth what it pretends to in a most admirable way. Not only are points of dissimilarity arranged in the most approved and intelligible way, but a marked feature of the work is the constant mention of "symptoms in common."

Whatever faults the work may contain are faults of omission mostly; though we are inclined to think that more space is given to the consideration of talipes than is necessary. In considering lesions about the hip-joint, we find no mention of the altered triangle formed by imaginary lines between the spine of the pubis, the anterior superior spine of the ilium, and the trochanter major — which seems an important oversight.

The nature of the work requires some repetition; but on the whole we are at a loss to see how more information could have been condensed in fewer words. While it is not complete, nor yet perfect in its present appearance, we regard it as a most valuable work, which ought to be found in every medical library.

R. P.

ARTICLE XXI. — GENERAL SURGICAL PATHOLOGY AND THERAPEUTICS, IN FIFTY-ONE LECTURES. By DR. THEODOR BILLROTH, Prof. of Surgery in Vienna. Translated from the fourth German edition with the special permission of the author, and revised from the eighth edition, by CHARLES E. HACKLEY, A.M., M.D., Physician to the New York Hospital, etc., etc. D. Appleton & Co., 1879.

This purports to be a revised edition of Dr. Hackley's translation of the fourth German edition of Billroth's Surgical Pathology; a work now too well and too favorably known to require more than a passing notice. The practical value of the "revision" is naturally lessened by the fact that much of the added matter is in the form of an "appendix," so that the reader is constantly annoyed by being obliged to fit little slices of matter into their appropriate places in the body of the book. The work is abundantly illustrated by wood cuts, most of which are conspicuously poor and coarse. If anything more absurdly impossible in the way of illustrative "diagrams" are to be found in any book printed within the last century, or if there are any "illustrations" in existence which more perfectly "illustrate" what "illustrations" should not be, than figs. 1, 2, 3, 4, 6 and 39 (which we cite as specimens merely), we have not chanced to see them. They remind me of the auction placards which adorn our fences and which are themselves adorned by amateur efforts in landscape gardening. But in spite of these faults, which ought to be corrected in the next "revision," Prof. Billroth's work is fairly entitled to be regarded as standard upon questions of surgical pathology. I. N. D.

ARTICLE XXII. — ELEMENTS OF COMPARATIVE ANATOMY. By Carl Gegenbaur, Professor of Anatomy and Director of the Anatomical Institute at Heidelberg. Translated by F. Jeffrey Bell, B.A., Magdalen College, Oxford. The translation revised and a preface written by E. Ray Lankester, M.A., F.R.S., etc., etc. London : Macmillan & Co., 1878.

We are compelled, both by want of space and want of time, to forego the pleasure of carefully reviewing this most excellent manual of comparative anatomy.

Perhaps the greatest merit of Prof. Gegenbaur's work consists in this — that "the comparative method is put prominently forward as the guiding principle in the treatment of the result of anatomical investigation," and this explains the real value of the study of comparative anatomy to the student of medicine.

We hope the time is not far distant when a course of practical lessons in comparative anatomy will take the place of the routine lectures on anatomy in the summer courses of instruction in our medical colleges. I. N. D.

ARTICLE XXIII. — AN INTRODUCTION TO PATHOLOGY AND MORBID ANATOMY. By T. Henry Green, M.D. Third American Edition. Philadelphia: H. C. Lea.

We are glad to welcome a new edition of this excellent manual of pathology. The work of revision has been carefully and thoroughly done. No student can afford or ought to be allowed to graduate without at least reading some work which embodies the essential doctrines of pathology ; and we know of no work which so fully meets the wants of the student and young practitioner. It is unfortunate that this author cannot, or does not, write a better chapter on the "cell," but he will probably get to it in the next edition. I. N. D.

ARTICLE XXIV.—PATHOLOGICAL REPORT OF THE MONTREAL GENERAL HOSPITAL, FOR THE YEAR ENDING MAY 1, 1877. BY WILLIAM OSTLER, M.D., of McGill Univeristy. Volume I. Montreal: Davison Brothers, 1878.

This little book of ninety-seven pages includes records of one hundred post mortem examinations, made in the "Montreal General Hospital." Dr. Ostler has the faculty of describing post mortem appearances clearly and tersely, and it is refreshing to get hold of a report of hospital work which is not burdened with wearisome details of "treatment" with which pathological reports are so often embarrassed. I. N. D.

